# Social and Emotional Functioning of Pediatric Brain Tumor Survivors and Typically Developing Youth Following the Onset of the Pandemic

**DOI:** 10.3390/curroncol31080324

**Published:** 2024-07-30

**Authors:** Leandra Desjardins, Kelly Hancock, Meng-Chuan Lai, Ute Bartels, Jacob Vorstman, Maru Barrera

**Affiliations:** 1 Charles-Bruneau Cancer Care Centre, Sainte-Justine University Health Centre, Montréal, QC H3T 1C5, Canada; 2 Department of Psychology, The Hospital for Sick Children, Toronto, ON M5G 1E8, Canada; 3 Department of Pediatrics, University of Montréal, Montréal, QC H3T 1J4, Canada; 4 Margaret and Wallace McCain Centre for Child, Youth & Family Mental Health, Azrieli Adult Neurodevelopmental Centre, and Campbell Family Mental Health Research Institute, Centre for Addiction and Mental Health, Toronto, ON M4V 1N6, Canada; 5 Department of Psychiatry, Autism Research Unit, The Hospital for Sick Children, Toronto, ON M5G 1E8, Canada; 6 Department of Psychiatry, Faculty of Medicine, University of Toronto, Toronto, ON M5T 1R8, Canada; 7 Division of Hematology/Oncology, The Hospital for Sick Children, Toronto, ON M5G 1E8, Canada

**Keywords:** pediatric oncology, social competence, emotional adjustment

## Abstract

**Background**: Social competence is a domain in which pediatric brain tumour survivors (PBTS) are at risk of challenges. To follow-up on our earlier work, in this study we assessed specific social interaction behaviors and emotional functioning in PBTS relative to typically developing youth (TD). The study coincided with the onset of the global pandemic. **Methods**: Sixteen PBTS and 16 typically developing youth (TD) between 8–16 years old participated in the study. Youth completed an assessment of social behavior and parents completed online surveys regarding child social and emotional adjustment. **Results**: PBTS experienced greater impairments in social interaction behaviors and on indices of social adjustment relative to TD. PBTS and TD experienced similar levels of emotional problems. Social behavior challenges were associated with indices of anxiety, rather than depression. Time since pandemic onset was not associated with social emotional outcomes. **Conclusions**: It will be important to monitor and support the social adjustment of populations such as PBTS, as well as the emotional adjustment across PBTS and TD youth, following the pandemic.

## 1. Introduction

Progress in medical treatments for pediatric brain tumors has led to a significant increase of survivors, and a pressing need to identify factors influencing their quality of life [1,2]. Over the past several decades, a growing body of research has highlighted problems in social competence as an important aspect of the psychosocial sequelae in pediatric brain tumor survivors (PBTS) [3,4,5]. Unfortunately, a significant impediment to the development of effective social competence interventions in PBTS is that, thus far, research on social competence in children with cancer has largely been based on broad, subjective (parent or child report) scales of social adjustment [4]. These measures assess the overall quality of a child’s social relationships, but provide limited insight into the specific social interaction behaviors that are considered to be key in influencing overall social adjustment, and which are also associated with cognitive and affective functioning [6]. Indeed, the social interaction behaviors of children with brain tumors have rarely been directly assessed. An obstacle to this line of inquiry is the shortage of observation measures of social interaction [7]. To address this knowledge gap, the current study aimed to build on pilot data from our team demonstrating the feasibility of using the Autism Diagnostic Observation Schedule Second Edition (ADOS-2) [8], a tool that was originally designed to support the diagnosis of autism, to assess and quantify discrete social behaviors in PBTS.

The pilot study found that the percentage of PBTS who experienced detectable social interaction impairments ranged from 0–50% across ADOS-2 items [9]. However, in the absence of data from typically developing (TD) participants, it is unclear which specific social behaviors PBTS experience impairment relative to that expected in TD youth, and thus which specific social behaviors should be the target of intervention. Thus, the primary aim of this study was to compare PBTS to TD for social behavior (assessed with the ADOS-2), social adjustment, and emotional functioning. The secondary aim was to examine associations between the ADOS-2 social behavior ratio score and measures of social, emotional, and cognitive functioning. PBTS are notably also at risk for elevated symptoms of depression and anxiety relative to healthy comparison youth [10] and youth with other cancers [11]. They are also at risk for cognitive late effects [12]. Both emotional and cognitive functioning are seen as influencing social behavior [6].

The initiation of this study coincided with the onset of the global COVID-19 pandemic, which presented significant challenges as well as unique opportunities to examine and compare PBTS and TD on social interaction skills, as well as social and emotional adjustment during this period. Previous research has highlighted both social and emotional challenges in PBTS [12] and that these domains are associated with each other [11]. Indeed, coping has been found to influence social adjustment in pediatric cancer [13]. Social interactions and support are essential to the psychosocial adjustment of children diagnosed with cancer [14]. The pandemic may have increased risk in both social and emotional domains of functioning, but there has been limited attention to PBTS as a vulnerable subgroup. One study found that the pandemic negatively impacted the social connectedness of PBTS, which has important implications for their emotional adjustment [15]. Currently, there is no information on how the pandemic affected the social behavior and social and emotional adjustment of PBTS compared to TD specifically. We hope this study may provide preliminary insights to fill this knowledge gap.

## 2. Methods

### 2.1. Participants

All study procedures were approved by the local hospital Research Ethics Board (REB #1000070921). PBTS youth were eligible to participate if they were 8–16 years of age, at least 1 year post-treatment for brain tumor diagnosis, and English speaking. Participants were excluded if the youth was actively receiving treatment for relapse or palliative care, if the youth had an existing diagnosis of autism or first-degree relatives with autism. Youths diagnosed with Tuberous Sclerosis Complex or Neurofibromatosis type 1 were also excluded, given the significantly increased likelihood of autism in these conditions [16,17]. TD youth were eligible to participate if they were 8–16 years of age and English speaking. TD youth were excluded if they had an existing autism diagnosis or first-degree relatives with autism.

Thirty-two youths (16 PBTS and 16 TD) consented to participate (see Table 1). Of the 16 PBTS who completed the ADOS-2: 56% were female, average age at enrollment was 13.24 years (standard deviation, SD 2.19, range 9–16), average age at diagnosis was 6.9 years (SD 4.74, range 1–13), and time since diagnosis was 6.35 years (SD 4.19, range 2–14). Diagnoses in the PBTS included 19% each of low-grade glioma, ependymoma, medulloblastoma, germinoma, and others (high-grade glioma, mixed germ cell, and ATRT). Medical treatment included resection only (6%), surgery and radiation (25%), surgery and chemotherapy (19%), and surgery, chemotherapy, and radiation (50%). Of the 16 TD, 75% were female, and the average age at enrolment was 14.81 years (SD 1.93, range 9–17).

### 2.2. Procedure

Eligible PBTS were identified via a patient database maintained by the neuro-oncology program at the institution and sent a letter describing the study and inviting them to participate. TD were identified by participating PBTS families (relatives or neighbors), advertising online, or through word of mouth. Potential TD were screened by phone by the project coordinator for eligibility. The overall assessment included an online and an in-person component. The former consisted of a RedCap query of youth demographics as well as proxy social adjustment and anxiety and depression measures, completed by caregivers within 1–14 days before the youth in-person assessment. The caregiver online measures took approximately 60 min to complete. The in-person assessment consisted of the ADOS-2 and cognitive measures, conducted within the outpatient psychology department of the hospital and took approximately 2 h to complete.

*Recruitment*. After receiving approval from the Research Ethics Board of a large pediatric oncology center in early March 2020, recruitment was delayed until November 2020. Although we initially planned to offer home assessments to support recruitment and convenience for families, these were not permitted for the duration of the study. While recruitment was initially planned for a year, it was extended due to the challenges of enrolling families because of the global pandemic. Thus, recruitment took place between November 2020 and March 2022. Sixty-six PBTS families were identified as eligible to participate in the study by the clinical neuro-oncology team. Of these, 6 could not be reached. This resulted in 60 potential PBTS families. Of these, 34 families declined participation stating they were too busy. Ten families declined because they did not want to come into the hospital solely for research during the pandemic. Sixteen PBTS families agreed to participate. PBTS were recruited 16 to 22 months after the pandemic onset and TD were recruited 19 to 24 months after the pandemic onset. Each participant, both youth and caregivers, received a $25 gift card.

### 2.3. Measures

Assessments occurred during the pandemic (16 to 24 months since pandemic onset). To comply with public health measures and hospital guidance in place at the time, ADOS-2 and cognitive assessments were performed with masks worn by both the participant and the tester in both groups. All institutional COVID-19 guidelines were followed. All materials were sanitized following each administration of the ADOS-2 as part of COVID-19 protocols.

*Social Behaviors*. The ADOS-2 is one of the most widely used instruments to quantify autism-related social communication and behavioral characteristics in a semi-structured, interactive context [18]. It is a reliable and well-validated standardized observational tool that consists of several modules [8]. The ADOS-2 targets the assessment of different aspects of social interaction skills (e.g., shared enjoyment, comments on others’ emotions, quality of social overtures, use of gestures, and eye contact). One of two trained assessors conducted the assessments using the ADOS-2 Module 3 or Module 4 [8]. The ADOS-2 Module 3 is intended for “Fluent Speech: Child and Adolescent” and Module 4 for “Fluent Speech: Adolescent and Adult”. Module 3 has some interactive play items that can be variably appealing to youth of younger ages. The assessor first determined which module was most appropriate for each youth. The two modules have the same coding items, except that Module 4 has three additional items. Data can be merged and compared across the intended ages. Of the 32 participants, 19 participants completed Module 4 (6 PBTS, 13 TD) and 13 participants completed Module 3 (10 PBTS, 3 TD). Each item on either module of the ADOS-2 is typically coded 0 (no or minimal impairment; developmentally appropriate behavior), 1 (some impairment), 2 (evident impairment), or 3 (evident and severe impairment).

*Social Skills*. Social skills were assessed by caregivers completing the Social Skills Improvement System (SSIS) online [19]. The SSIS provides an age- and sex-normed total standard score representing four subscale scores: cooperation, assertion, self-control, and responsibility. The SSIS has adequate reliability and validity and, compared with other measures used to assess social competence, has the most comprehensive data on pediatric brain tumor survivors [20]. Total score reliability coefficients are typically greater than 0.90 and subscales greater than 0.70 [19]. Lower scores on the total scale reflect greater problems in social skills.

*Social and Emotional Adjustment*. Caregivers completed the Child Behavior Checklist online (CBCL; Ref. [21]. Reliability and validity are well established for the CBCL, and normative T scores are derived from caregivers’ reports on a nationally representative sample of youth ages 6–18 years old. Social adjustment was assessed via the Social Problems and Social Competence subscales. Emotional adjustment was assessed via the Anxious Depressed, Withdrawn Depressed, Anxiety Problems subscales and Internalizing Problems scale. Subscale reliability coefficients typically range from 0.71–0.89, and convergent validity correlations with other parent measures range from 0.54–0.59 [22].

*Cognitive Functioning*. Participant IQ was estimated using two subtests of the Wechsler Abbreviated Scale of Intelligence, Second Edition (WASI-II): Vocabulary and Matrix Reasoning [23]. The WISC-V Coding and Digit Span subtests were also administered to obtain measures of processing speed and working memory, respectively [24]. The internal consistency reliability for composite, subtest, and process scores typically ranges from *r* = 0.80 to *r* = 0.96 [25].

*Medical and Demographic Information*. Youth medical variables examined were: tumor type, age at diagnosis, time since diagnosis, and treatment. Youth age and sex were included as demographic factors.

### 2.4. Data Analysis

Preliminary analyses compare age, sex, and ADOS-2 Module 3 versus 4 used in PBTS versus TD groups. Independent samples T-test was used for continuous data (age) and non-parametric chi-square analysis was used for dichotomous data (sex and Module). We also examined correlations between time between pandemic onset (in months) and measures of social and emotional functioning. Consistent with the pilot study (Desjardins et al., 2021 [9]), we created an ADOS-2 “overall score” by first summing the original scores (0–3) across all ADOS-2 individual items, then dividing the sum by the maximum for each Module to account for the three additional items in Module 4 relative to Module 3 (i.e., 32 items in Module 4 and 29 items in Module 3). Given the presence of masking during the assessment, we did not retain the item “facial expressions” typically coded by the ADOS-2. An independent sample T-test was used to compare groups on overall ratio scores. To identify specific aspects of atypical behaviors, each of the ADOS-2 individual item scores were dichotomized as “0” versus “1–3”, with 0 indicating the absence of impairment and 1–3 indicating the presence of at least some impairment in the particular behavior. The percentage of PBTS or TD participants having at least some impairment on each ADOS-2 item is reported descriptively. Consistent with the pilot study, we also note items where >40% of the sample experienced impairment. In addition, the PBTS and TD groups were compared using independent samples t-tests on the social, emotional, and cognitive measures. Finally, exploratory bivariate correlations were performed between the ADOS-2 social behavior ratio score and social, emotional, and cognitive functioning variables. Given the limited sample size, no corrections were performed for multiple comparisons. Consequently, all statistical analyses conducted are considered exploratory.

## 3. Results

### 3.1. Preliminary Analyses

Descriptive statistics are presented in Table 1. The sex distribution was similar (*p* > 0.05) for PBTS (44% male) and TD (25% male). There was a difference in age between groups (*t*(30) = −2.14. *p* < 0.05), with TD being slightly older (M = 14.81, SD = 1.93) than PBTS (M = 13.24, SD = 2.19). Correspondingly, there were a greater number of Module 4 ADOS-2 assessments in TD (n = 13) versus PBTS (n = 6) (*X*^2^ = 6.35, *p* < 0.05). The number of months since the pandemic onset was not significantly correlated with any social or emotional variables (*r*s: 0.04 to 0.22; all *p*s > 0.20).

### 3.2. Descriptive Statistics of Specific Social Behaviors on the Modified ADOS-2 in PBTS vs. TD

**Figure 1:**
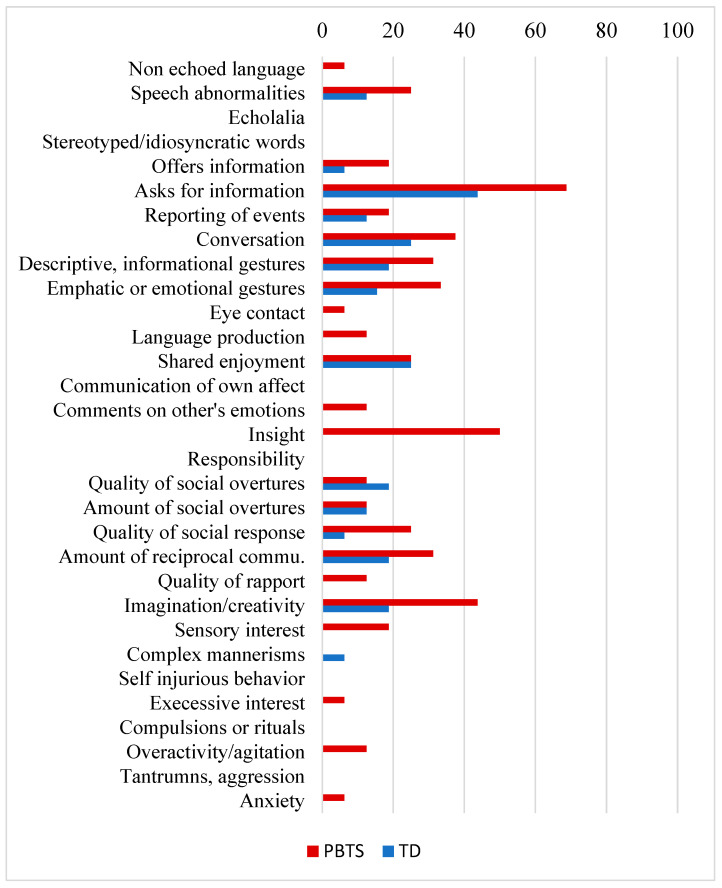
Specific Social Behaviors Coded by the ADOS-2 in PBTS vs. TD.

The overall ADOS ratio score was higher in PBTS (M = 0.09; SD = 0.10) relative to TD (M = 0.03; SD = 0.04; *t* = 1.91, df = 30, d = 0.68, *p* < 0.05). Rates of impairment ranged from 0–69% for PBTS and 0–44% for TD across individual ADOS-2 items (see Figure 1). Impairments were captured across 75% of ADOS-2 items (24/32) for PBTS and 44% of ADOS-2 items (14/32) for TD. Within PBTS, there were three items for which more than 40% of PBTS experienced difficulty: Asking for Information (69%), Insight into Typical Social Situations and Relationships (50%), and Imagination/creativity (44%). Within TD, there was only one item for which more than 40% of the sample struggled: Asking for Information (44%). For both PBTS and TD, 0% of the sample were found to experience challenges on individual social behavior items such as: Echolalia, use of Stereotyped/Idiosyncratic words, Complex mannerisms, Self-injurious behavior, Compulsions or rituals, Tantrums.

### 3.3. Social and Emotional Adjustment and Cognitive Functioning in PBTS vs. TD (See Table 2)

PBTS obtained lower scores relative to TD across social adjustment indices (SSIS total social skills score, CBCL Social Problems and Social Competence subscales; all *p*s < 0.05; see Table 2). There were no significant differences between PBTS and TD across emotional adjustment indices (CBCL Anxious Depressed, Withdrawn Depressed, Anxiety Problems, Internalizing Problems subscales; all *p*s > 0.05).

**Table 2:** Social, emotional, and cognitive functioning indices in PBTS and TD.

	PBTS		TD		*t*	d
	M (SD)	Range	M (SD)	Range		
Social Skills (SSIS)	99.38 (19.31)	70–129	112.63 (13.68)	89–136	−2.24 *	−0.79
Social Problems (CBCL)	56.44 (8.78)	50–75	51.13 (2.13)	50–58	2.35 *	0.83
Social Competence (CBCL)	44.44 (9.01)	31–65	53.81 (6.41)	41–65	−3.39 *	−1.20
Anxious Depressed (CBCL)	57.19 (7.96)	50–84	55.81 (9.77)	50–84	0.44	0.15
Withdrawn Depressed (CBCL)	56.25 (7.67)	50–70	55.38 (6.15)	50–70	0.36	0.13
Anxiety Problems (CBCL)	58.63 (8.18)	50–77	54.38 (7.83)	50–72	1.50	0.53
Internalizing Problems (CBCL)	53.81 (10.06)	33–76	49.63 (12.52)	33–76	1.04	0.37
Full Scale IQ-2 (WASI-II)	100.13 (25.70)	51–129	113.31 (10.29)	92–135	−1.91 *	−0.67
Working Memory (WISC-V)	11.25 (4.91)	4–19	11.81 (3.27)	7–19	−0.38	−0.14
Processing Speed (WISC-V)	6.87 (4.53)	1–16	11.38 (3.74)	7–19	−3.03 **	−1.09

Note. * *p* < 0.05; ** *p* < 0.01.

We also compared the cognitive functioning between PBTS and TD. PBTS obtained lower scores in overall IQ on the WASI-II and processing speed on the WISC-V (both *p*s > 0.05), but did not significantly differ in scores on the working memory subscale of the WISC-V (*p* > 0.05). Note. * *p* < 0.05; ** *p* < 0.01.

### 3.4. Associations between Social Behaviour Ratio Score, Social and Emotional Adjustment, and Cognitive Functioning

Within the full sample, bivariate correlations of social behaviour ratio score and social, emotional adjustment, and cognitive functioning indicated that social behavior challenges on the ADOS-2 were associated with lower social skills, higher social problems, internalizing problems, higher scores on anxiety-related scales (Anxious Depressed, Anxiety Problems), lower overall cognitive functioning, and lower processing speed (see Table 3). A similar correlation pattern was found within the PBTS group, with significant associations only for internalizing problems and specific anxiety problems. While the associations with internalizing problems and social behavior followed a similar pattern for the TD group, none were significant (see Table 3).

## 4. Discussion

The global COVID-19 pandemic had a significant impact on the social context youth were exposed to, and the short and long-term effects of this event warrant study. On a broader level, the pandemic also disrupted many research projects during this time, as we all learned to adjust to this changing environment [26]. Indeed, our own initially planned study was modified in the context of the pandemic, with a longer recruitment window, a smaller sample size than anticipated, and the inclusion of masking and social distancing procedures during direct testing. Here we share the results of our study which aimed to examine the specific social interaction behaviors of PBTS and TD, as well as their broader social, emotional, and cognitive functioning following the onset of the pandemic.

Overall, compared to TD, PBTS showed impairments in the ADOS-2 overall ratio score and questionnaire measures of social competence. The ADOS-2 social behavior items where PBTS appeared to experience the most challenges included Asking for Information, Insight into Typical Social Situations and Relationships, and Imagination/Creativity. These social behavior findings confirm previous work with a pre-pandemic sample of PBTS [9]. The only ADOS-2 item for which more than 40% of both PBTS and TD appeared to experience challenges was Asking for Information. Challenges in requesting information may have implications for peer social interactions, specifically for PBTS in contexts such as medical follow-up appointments. Greater challenges on the ADOS-2 were unsurprisingly associated with lower social skills and greater social problems. Across PBTS and TD, higher indices of anxiety symptoms and lower overall IQ and processing speed were each associated with more social interaction behavior challenges. This finding is in line with social competence models that indicate that cognitive and affective processes influence youth social behavior [6]. The impact of anxiety symptoms on social behavior appeared especially pronounced in PBTS, highlighting the need to attend to social and emotional functioning in concert, especially following significant unpredictable and uncontrollable stressors (e.g., the pandemic, brain tumor diagnosis, and treatment). However, these findings need further validation with a larger sample.

With regards to social adjustment, social skills scores (SSIS), social problems, and social competence scores (CBCL) suggest social adjustment deficits in PBTS compared to TD. These findings are consistent with previous studies highlighting social competence as an area of difficulty for PBTS [3,4]. Thus, while these differences were likely pre-existing to the pandemic, the pandemic experience may have exacerbated this effect. It is possible that children with pre-existing friendships were able to maintain these via virtual communication means, while it may have been more difficult to create new relationships during the social interaction restrictions of the pandemic. It will be important to monitor over time and pay attention to PBTS social relationships, particularly post-pandemic and with reintegration of social interactions without restrictions. With regards to cognitive functioning, Full-scale IQ and processing speed scores suggest greater cognitive challenges in PBTS compared to TD. These cognitive findings are similar to those of the pilot study, and reflect those of other studies, suggesting that while the sample in this study is at least in some ways comparable to PBTS recruited pre-pandemic [9], further investigation with a larger sample is needed.

Emotional adjustment indices (depression and anxiety scores, and internalizing problems, CBCL) were generally similar across groups. Notably, the scores of both groups were slightly elevated compared to the normative mean of 50, indicating the presence of symptoms of anxiety and depression. These findings are coherent with reports that the mental health of children was negatively impacted during the pandemic [27]. The fact that the groups did not differ in anxiety or depression scores is also consistent with other studies finding no differences in psychosocial functioning in the pediatric cancer population relative to healthy controls [28]. Indeed, the pandemic may have had an “equalizing effect”, exposing PBTS and TD similarly to a global stressor, which significantly impacted their well-being. All youth were subject to social interaction restrictions, possible school closures, changes in family routines, and the stress of exposure or possibly to the virus. Thus, all youth experienced risk factors for increased challenges in social and emotional functioning.

*Implications for practice*. These findings highlight the need to attend to both PBTS and TD’s social and emotional well-being following the pandemic. Difficulties in asking for information seen across youth are important to consider in the context of medical care. Being able to ask for information during medical appointments is an essential skill for youth to develop over time, particularly as they prepare to transition from pediatric to adult care [29]. Resources exist to help develop asking for information as a skill, including tools that can screen for this skill [30] and also websites with examples of questions youth can ask their care providers (https://www.connecticutchildrens.org/growing-healthy/24-questions-your-teen-ask-their-doctor, accessed on 14 September 2023). More recently, an intervention for PBTS has included teaching youth how to prepare questions for their medical team as an intervention target [31]. Furthermore, findings from this study highlight the need to monitor depression and anxiety symptoms and support coping with uncontrollable stressors across children [32]. Challenging global contexts such as pandemics and wars may increase the risk of distress among children. Pediatric cancer is associated with many additional uncontrollable stressors [33]; therefore, PBTS may be especially vulnerable. Screening for distress in clinical care offers an opportunity for equitable and early identification and intervention. Screening for depression and anxiety is recommended across all children [34,35], and psychosocial screening is a standard of care in pediatric oncology [36].

*Study Strengths*. This is the first study that used a healthy control group as a reference for investigating the social behaviors of PBTS using the ADOS-2. These findings provide a foundation for further examination of the strengths and deficits in social behavior, social adjustment, and mental health in PBTS compared to TD with a larger sample to determine the critical intervention targets for PBTS. In addition, the use of a combination of direct observation, performance-based measures, and subjective reporting of social behavior and adjustment, cognitive, and mental health represent a rich dataset serving to further our understanding of the complex nature of PBTS survivorship. In addition, while it is important to share major scientific research breakthroughs, it is equally important to share major research challenges, particularly when the challenges are related to a global pandemic. Our study documents the impact the COVID-19 pandemic had on recruitment and the challenges for assessment during this period.

*Study limitations and future directions*. Several potential limitations of this study need to be considered. The pandemic significantly impacted study recruitment, which resulted in a much smaller sample than anticipated, limiting the power of analyses. Second, participants who agreed to participate in a study related to social competence during the pandemic may differ from those who declined to participate. Therefore, these preliminary findings should be interpreted with caution. There were some assessment modifications, including the use of masking during the in-person assessment of youth in both groups. This may have hindered some potential non-verbal communication in the lower-mid part of the face. Therefore, we did not retain the item “facial expressions” typically coded within the ADOS-2. At this point in the pandemic, communicating while using masks had been integrated into many social situations (ex., school, medical appointments), thereby replicating many real-world social interactions during this period.

## 5. Conclusions

Overall, the global COVID-19 pandemic had a significant impact on the social context youth were exposed to, and the short and long-term effects of this event warrant further study. On a broader level, the pandemic disrupted many research projects, as we all learned to adjust to this changing environment [26]. Indeed, in our study, we made modifications in the context of the pandemic, including changes in the recruitment process and duration, and the inclusion of masking during direct testing. Even with these adjustments, we recruited a smaller sample size than anticipated. Despite these challenges, the results of our study have provided some insights into the social competence and emotional functioning of PBTS relative to TD during this time, and raise further questions for future research, including a need to monitor and support the social and emotional functioning of both PBTS and TD youth over time following the pandemic.

## Figures and Tables

**Table 1:** Participant characteristics.

	Brain Tumor (n = 16)	Typically Developing (n = 16)
Variables	n (%) or M ± SD	n (%) or M ± SD
Age in years	13.24 ± 2.19	14.81 ± 1.93
Female sex	56%	75%
Race		
White	8 (50)	7 (44)
Black		2 (13)
Asian	5 (31)	4 (25)
Multiracial	3 (19)	3 (19)
Family income		
Under 25,000 per year	4 (25)	
500,000–75,000 per year	5 (31)	2 (13)
75,001 to 100,000 per year	3 (19)	5 (31)
100,001 or more per year	4 (25)	8 (50)
Prefer not to say		1 (6)
Time since diagnosis in years	6.35 ± 4.19	
Tumour type		
Medulloblastoma	3 (19)	
Low grade glioma	3 (19)	
Ependymoma	3 (19)	
Germinoma	3 (19)	
Other (Astrocytoma, mixed germ cell, ATRT)	4 (24)	
Tumour Treatment		
Surgery only	1 (6)	
Chemotherapy and surgery	3 (19)	
Radiotherapy and surgery	4 (25)	
Chemotherapy, radiation, and surgery	8 (50)	

**Table 3:** Correlations between social behavior ratio, social and emotional adjustment, and cognitive functioning.

	SS	SP	SC	AD	WD	AP	IP	FSIQ	WM	PS
Full sample ADOS-2 overall ratio score	−0.36 *	0.49 **	−0.25	0.37 *	0.27	0.48 **	0.45 *	−0.50 **	−0.27	−0.41 *
PBTS	−0.23	0.44	0.003	0.41	0.33	0.52 *	0.54 *	−0.49	−0.34	−0.35
TD	−0.41	0.21	−0.27	0.42	0.25	0.28	0.45	−0.16	0.01	−0.26

Note. SS = Social Skills (SSIS), SP = Social Problems (CBCL), SC = Social Competence (CBCL), AD = Anxious Depressed (CBCL), WD = Withdrawn Depressed (CBCL), AP = Anxiety Problems (CBCL), IP = Internalizing Problems (CBCL), FSIQ = Full Scale Intellectual Quotient (WASI), WM = Working Memory (WISC), PS = Processing Speed (WISC). * *p* < 0.05; ** *p* < 0.01.

## Data Availability

The data presented in this study is available on request from the corresponding author.
